# A mouse model to study infection against porcine circovirus type 2: viral distribution and lesions in mouse

**DOI:** 10.1186/1743-422X-7-158

**Published:** 2010-07-15

**Authors:** Jun Li, Xiaoyuan Yuan, Chaofan Zhang, Lanfei Miao, Jiaqiang Wu, Jianli Shi, Shaojian Xu, Shangjin Cui, Jinbao Wang, Hongbin Ai

**Affiliations:** 1 College of Life Sciences, Key Laboratory of Animal Resistance of Shandong Province, Shandong Normal University, Jinan, 250014, China; 2 Division of Swine Diseases, Shandong Provincial Key Laboratory of Animal Disease Control & Breeding, Institute of Animal Science and Veterinary Medicine Shandong Academy of Agricultural Sciences, Jinan, 250100, China; 3 Division of Swine Infectious Diseases, State Key Laboratory of Veterinary Biotechnology, Harbin Veterinary Research Institute of Chinese Academy of Agricultural Sciences, Harbin, 150001, China

## Abstract

**Background:**

Little information is known about viral distribution and transmission of porcine circovirus type 2 (PCV2) in species other than swine. It is still a debated topic whether the PCV2 could be infected and caused clinical lesions. Our study is aimed to estimate the susceptibility of Kunming mouse to PCV2. Forty-eight, 6-week-old Kunming mice were randomly divided into four groups. Group A (C1-C12) was inoculated with PK-15 cell culture as a control group. Group B (sPCV1-12) was inoculated orally and intramuscularly with PCV2 (10^6.2^TCID_50_/ml). Group C (mPCV1-12) was inoculated orally and intramuscularly with PCV2 (10^6.2^TCID_50_/ml) and a booster inoculation at days 14 and 28 after the first inoculation. Group D (MixPCV1-12) was unvaccinated but released into Group C. Each group was sacrificed at 7, 14, 28, and 42 days post-inoculation, respectively. Necropsy was checked on every mouse. Sera samples were collected for the test of PCV2 specific antibody. Tissues were collected for histopathology study and polymerase chain reaction (PCR).

**Results:**

The results showed that viral replication, seroconversion, and microscopic lesions were found in inoculated mice. Continuous existence of PCV2 viruses in lymph nodes have been confirmed by PCR, which took at least seven days for the virus to be transferred into other organs from the primary interface, and the diffusion to thymus had been retarded for seven days. Special PCV2 antibody could be found in PCV2 inoculation mice and was significantly higher than that in the control. Further more, microscopic lesions and the main target of PCV2 focused in the lymph nodes with a characteristic depletion and occasional necrosis of lymphocytes in the cortex and paracortex were found in inoculated mice.

**Conclusions:**

The Kunming mouse could be infected by PCV2 virus and used as a PCV2 infected experimental model.

## Background

Porcine Circovirus (PCV), a member of genus *Circovirus *of the *Circoviridae *family, was first isolated as a non-cytopathic contaminant of a porcine kidney cell line (PK-15) and has been characterized as a small icosahedral DNA virus [1-3], which was the primary causative agent of an emerging swine disease- postweaning multisystemic wasting syndrome (PMWS) [4]. The clinical signs were characterized by progressive weight loss, dyspnea, tachypnea and icterus in post-weaned pigs of approximately 8-12 weeks of age [5]. Gross lesions in pigs with PMWS consist of generalized lymphadenopathy in combination with less frequent lesions in the lungs, liver, kidneys and stomach [6]. The most consistent microscopic lesions in affected pigs are in lymphoid organs and include lymphoid cell depletion and glaucomatous inflammation with inconsistently occurring intracytoplasmic viral inclusion bodies in macrophages.

Recently, PCV2 disease has become a major immuno-suppression problem for large-scale pig farms and caused a great economic loss worldwide [7]. But, it is still difficult to copy the clinical and pathologic features of PMWS in lab. Clinical PMWS had been reproduced in gnotobiotic pigs co-infected with PCV2 and porcine parvovirus (PPV) [5,8], however, no clinical PMWS found in gnotobiotic pigs for just being infected with PCV2 alone [8,9]. Whether PCV2 can infect mice or other mammalian species is still a debated topic. Kiupel [10] succeed in an experimental model in BALB/c mice, but Quintana [11] indicated that the PCV2 can't replicate in mice. The aim of this study was to make sure whether PCV2 could replicate and distribute in Kunming mouse.

## Results

### Distribution of PCV2 in different organs clarified by polymerase chain reaction

The fresh tissues of heart, liver, spleen, lung, kidney, thymus, lymph node, jejunum, ileum, cecum, colon, tongue and brain of each mouse were supplied for PCR. As illustrated in Table 1, at day 7, the PCV2 was detected in each tissue of sPCV and MixPCV mice except thymus, tongue and brain. At day 14, the virus could be detected in thymus, but the kidney was negative. The PCR results of PCV2 in other tissues were the same to that of day 7. At day 28, the virus could only be found in the thymus and lymph node. At day 42, PCV2 still could be found in the lymph node while its existence in other tissues was not obvious. The cPCV mice were negative, thoughout of the experiment. The above data implied that there was viral replication in the PCV2 inoculation mouse groups.

**Table 1:** Distribution of PCV2 in sPCV at Different Time

Time	Heart	liver	spleen	lung	kidney	thymus	lymph node
day 7	+++	+++	+++	+++	+++	-	+++
day 14	+++	+++	+++	+++	-	++	+++
day 28	-	-	-	-	-	+++	+++
day 42	-	-	-	-	-	-	+++

+: one positive result, ++: two positive results, +++: three positive results, -: negative result

### The results of necropsy

Throughout the experiment, all of the mice survived under the PCV2 inoculation and no clinical syndrome was observed on cPCV, sPCV, or MixPCV mice. No gross lesion was found in cPCV, sPCV, or MixPCV mice. In contrast, 8 of 12 mPCV mice had obvious intumesce in the lymph node. 1 of 12 mPCV mice had obvious intumesce in the spleen. There were no other lesions found in other tissues.

### Results of seroconversion antibody to PCV2

**Figure 1:**
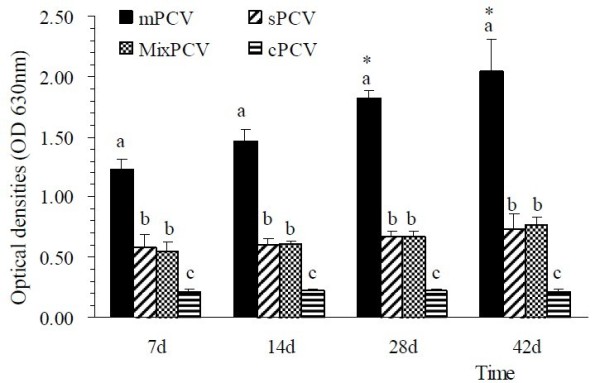
**Identification of special antibody of PCV2 for cPCV, sPCV, mPCV and MixPCV mice**. X-axis represents the value at optical densities (OD630nm), Y-axis represents the days PI. Different letters "a", "b", and "c" represent significance. The level of significance was set at P < 0.05.

The anti-PCV2 special antibody level was evaluated by ELISA. As shown in Figure 1, at day 7, seroconversion to PCV2 can be found in 3 of the mice sacrificed in all PCV2 inoculation groups and the MixPCV group. The special antibody level was steady for 42 days at least. In mPCV mice, the antibody level was significantly higher than that in sPCV mice and MixPCV mice (*P <*0.05). The special antibody level of sPCV mice and MixPCV mice was significantly higher than that in cPCV mice (*P <*0.05). Antibody levels of all PCV2 inoculation groups and the MixPCV group were significantly higher than that in cPCV mice (*P <*0.05). In contrast, only background levels of antibody responses were detected in cPCV mice. The results implied that special PCV2 antibody can be found in PCV2 inoculation mouse groups.

### Histopathological results

**Figure 2:**
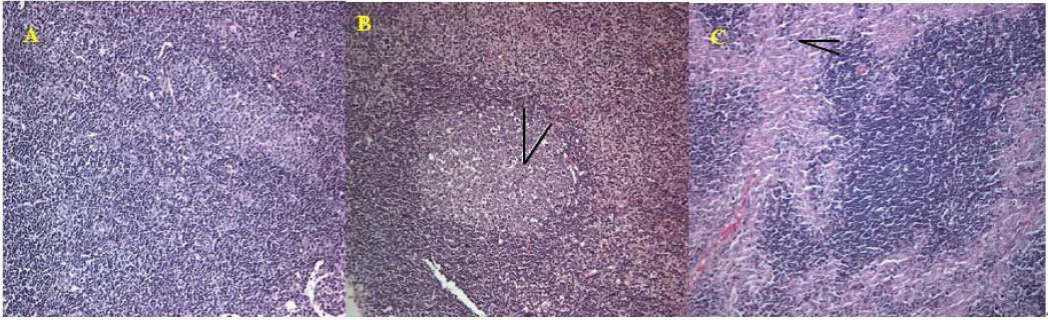
**Pathological section for lymph node**. A: Normal lymph node. B: Lymph node from sPCV-inoculated mouse, 28 days PI. Necrosis for lymphoid nodule and present vacuolus (arrows). C: Lymph node from mPCV-inoculated mouse 42 days PI. Necrosis for lymph node and low-grade fibrosis (arrows). HE staining, ×200.

The lesions of PCV2 infection were concentrated in the lymph node. As shown in Figure 2, apparent necrosis of lymphocyte could be found in the lymphaticus of sPCV mice, while some of the lymphocytes disappeared with a vacuolus remaining. In mPCV mice, the necrosis of lymphocyte was more serious than those in sPCV mice. Some of the lymphocyte was substituted by fibrosis.

**Figure 3:**
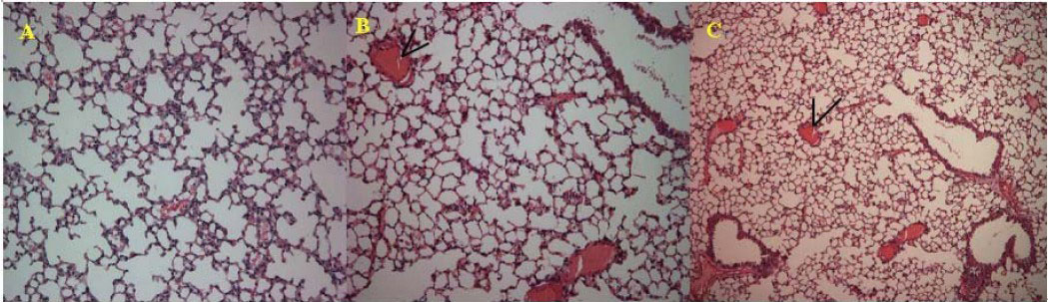
**Pathological section for lung**. A: Normal lung. B: Lung from sPCV-inoculated mouse 28 days PI. Thrombus for lung (arrows). C: Lung from mPCV mouse, thrombus and inflammation in veins. HE staining, ×200.

**Figure 4:**
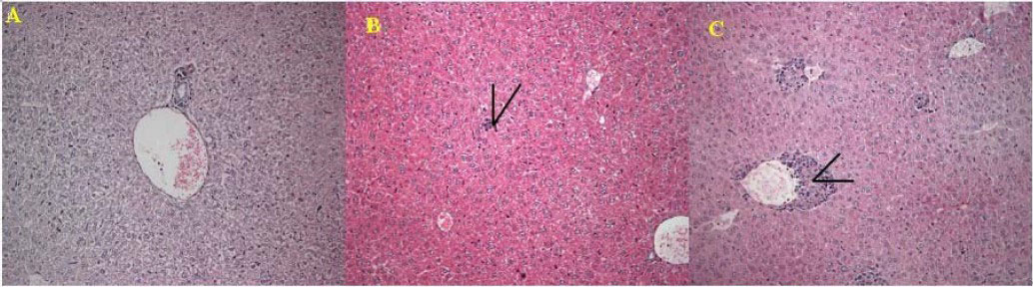
**Pathological section for liver**. A: Normal liver. B: Liver from sPCV mouse, infiltration of lymph cells. C: Liver from mPCV-inoculated mouse 42 days PI. Necrosis, infiltration of lymph cell, and severe vasculitis for liver (arrows). HE staining, ×200.

**Figure 5:**
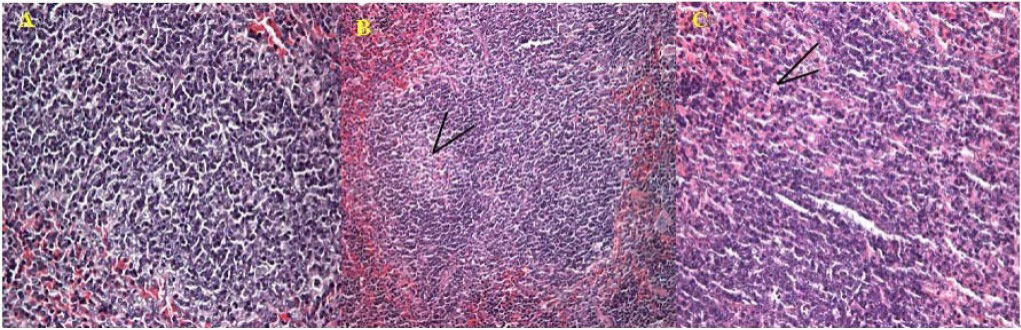
**Pathological section for spleen**. A: Normal spleen. B: Spleen from sPCV mouse, necrosis and reduction of lymph cells in lymph nodule. C: Spleen from mPCV-inoculated mouse, necrosis of lymph cells and low-grade disintegration (arrows). HE staining, ×400.

In all of the PCV2 inoculated mice, the structure of alveolus was obvious. No collapse or exudation was found in the alveolus cavity, but thrombus was deposited on the walls of veins in sPCV and mPCV groups (Figure 3). There was infiltration of lymph cells and severe vasculitis of liver in mPCV mice. In contrast the inflammation was not very serious, with only slight infiltration of lymph cells for liver in sPCV mice (Figure 4). In the spleens of sPCV mice, the necrosis and absence of lymph cells in lymph nodes was found. In mPCV mice, the disintegration of lymph cells can be seen in the spleen (Figure 5). There was no obvious change in other tissues, so the figures were not attached. The above data implied that PCV2 could cause lesions on the Kunming mouse and pathological changes were focused in the lymphatic organ, spleen, lung and liver.

## Discussion

To date, limited information was available on the host range of PCV2, but antibodies were found in various species other than pigs, including humans, mice, and cattle [11,12]. Whether PCV2 can cause clinical lesions in a mouse is still a debated topic. Therefore, Kunming mouse were chosen as the experimental animal for our research. Results of this study demonstrate that PCV2 can replicate in Kunming mouse.

Kiupel [10] demonstrated that PCV2 was capable of replicating in BALB/c mice and caused microscopic lesions, which were similar to that of PCV2 infection in pigs. In contrast, Quintana [13] reported that no microscopic lesions compatible with PCV2 infections were detected in inoculated mice, but it was considered to be due to the dosage of the inoculums and administration route. The multi-route and large doses of injection were adopted in this experiment. In this study, viral replication, seroconversion, and microscopic lesions were characterized in inoculated mice. Continuous existence of virion in the lymph node has been confirmed by PCR. Necropsy results shown that 66.7% mPCV mice had obvious intumesce in the lymph node. One mPCV mouse had obvious intumesce in the spleen. There were no other lesions found in other tissues. The results are as well as those obtained by Kiupel [10], who found some evidence of PCV2 replication in tissues of BALB/c mice. The same conclusion can be replicated in different mice with different PCV2 isolates and viral inoculation as in this study (date not shown).

The ability of PCV2 to infect [4] and cause lesions in pigs was demonstrated previously in experimentally infected pigs [14]. In affected lymph nodes from inoculated piglets, marked expansion of cortical and paracortical zones, with infiltration by cells of monocyte/macrophage morphology, was found. Depletion and occasional necrosis of lymphocytes is a characteristic result after PCV2 infection. In this study, we also found that the distribution and lesions of PCV2 infection has an obvious regularity. First, it has taken at least 7 days for the PCV2 to be transferred into other organs from the primary interface. It seems that the diffusion to thymus had been retarded for 7 days. The replication of PCV2 can be continually detected in the lymph node until the end of the experiment, which was in accord with those by the piglets. Second, we found that PCV2 could make microscopic lesions on the mouse such as lymphatic organ, spleen, lung and liver. The main target of PCV2 in the mouse was focused in the lymph node, with a characteristic depletion and occasional necrosis of lymphocytes in the cortex and paracortex. Overall in mPCV mice, microscopic lesions were more serious than those in sPCV mice. There was infiltration of lymph cells and severe vasculitis of liver in mPCV mice. In contrast the inflammation was not very serious, with only slight infiltration of lymph cells for liver in sPCV mice. In the spleens of sPCV mice, the necrosis and absence of lymph cells in lymph nodes was found. In mPCV mice, the disintegration of lymph cells can be seen in the spleen. We could get a conclusion that PCV2 could cause lesions in Kunming mouse which were similar to that of Kiupel reported[10] and more and more seriously with the PCV2 increasing. However, the mechanism of PCV2 induced damage on lymphocytes and spleen is not clear, it was need to test in further study and discussion.

The anti-PCV2 special antibody can be found in PCV2 inoculation mouse groups and was steady for 42 days at least. The results of seroconversion antibody to PCV2 were another evidences to the conclusion that PCV2 could replicate in Kunming mouse.

## Conclusions

In summary, viral replication, seroconversion, and microscopic lesions have been determined in inoculated mice, and the persistent existence of PCV2 in the lymph were also confirmed by PCR, which suggest that the Kunming mouse can be infected by PCV2, and used as a PCV2 infected experimental model.

## Materials and methods

### Virus, Cells and cell culture

The PCV2 virus strain SD2(DQ478947) was propagated in PCV1-free porcine kidney 15 (PK-15) cell lines, maintained in RPMI medium 1640 supplied with 10% fetal calf serum. The inoculums contained 1 × 10^6.2 ^infectious viruses per millilitre when titrated on a PCV-free PK-15 cell monolayer.

### Animal experiment

Forty-eight 6-week-old Kunming mice were purchased from the experimental animal centre of Harbin Veterinary Research Institute of Chinese Academy of Agricultural Sciences. Mice were maintained in isolation rooms in filter top cages and any handling and husbandry procedures were performed under a laminar flow hood. The experimental animals were randomly divided into four groups. Group A (cPCV1-12) was inoculated with PK-15 cell culture of RPMI medium 1640 at days 1, 14, and 28 post inoculation as the control group. Group B (sPCV1-12) was inoculated orally and intramuscularly with 0.1ml PCV2 (10^6.2^TCID_50_/ml) at day 1. Group C (mPCV1-12) was inoculated orally and intramuscularly with 0.1ml PCV2 (10^6.2^TCID_50_/ml) at day 1 and with a booster inoculation at days 14 and 28 post first inoculation. Group D (MixPCV1-12) was unvaccinated but released into Group C at day 1. The condition of the mice was recorded every day.

### Necropsy and histopathological analysis

Three mice from each group were killed by bleeding at days 7, 14, 28, and 42 respectively and the sera were collected. Any gross lesions at necropsy were recorded. Fresh heart, liver, spleen, lung, kidney, thoracic gland, lymph node, dodecadactylon, jejunum, ileum, caecum, colon, rectum, tongue and brain of each mouse were collected. Tissues with evident lesions were fixed in 10% neutral buffered formalin. Fixed tissues were dehydrated in a series of alcohols, cleared in xylene, and embedded in paraffin. Sections were stained with hematoxylin and eosin and then were observed under a light microscope.

### Detection of PCV2 by polymerase chain reaction

A pair of primers was designed based on the published sequences of PCV2 (upstream primer, 5'-AAGGGCTGGGTTATGGTATG-3'; downstream primer, 5'-CGCTGGAGAAGGAAAAATGG-3').

The genome DNA of PCV2 was extracted with a previously reported method [15]. After being freezed and thawed three times, 0.1 g of the tissues (heart, liver, spleen, lung, kidney, thymus, lymph node, jejunum, ileum, cecum, colon, tongue and brain) were homogenized with a homogenizer. 500 μL of the samples were digested with 1 μL of proteinase K (Sigma) and then incubated at 50°C for 1.5 hours. The digestion samples were extracted with equal volume of phenol-chloroform (1:1 v/v). After centrifuged at 12,000g for 15 minutes, the upper layer was transferred to new Eppendorf tubes. 200 μL of isopropanol was added to each tube, which were then incubated at -20°C for 1 hour. This was followed by centrifugation at 12,000 g for 10 minutes. Then the supernatant was discarded and the pellet was washed once with 75% ice-cold ethanol, after which it was dried in a laminar flow cabinet. The precipitate of DNA was dissolved in 50 μL of sterile water and then stored in -20°C for later use.

PCR was carried out in a 50 μL reaction volume containing 4 μL of dNTP mixture (2.5mmol/L), 5 μL of 10× PCR buffer, 5 U of Taq polymerase (TaKaRa Company), 10 μM each of primers, 33.5 μL H_2_O and 1 μL of precipitate DNA extracted previously. PCR was performed as follows: 94°C for 5minutes followed by 30 cycles of 94°C for 45 seconds, 56°C for 30 seconds, and 72°C for 30 seconds, with a final extension step for 7 minutes at 72°C. The PCR was carried out in the 2720 Thermal Cycler (Applied Biosystems). PCR products were subjected to electrophoresis on a 1% agarose gel.

### Enzyme-linked immunosorbent assay for antibody level

The sera from experimental animals were handled according to a previous reported method [10]. Sera collected from cPCV, sPCV, mPCV, and MixPCV mice were tested for special antibodies to PCV2 by Enzyme-linked immunosorbent assay (ELISA). A commercial ELISA kit bought from Green Spring Biotechnology Company was used in this study. 100 μL of diluted samples (1:40, v/v) were added into each well and the plate was incubated at 37°C for 30 minutes. After shaking out the samples, washing each plate six times with 1× TBST, and slapping the plate face down onto a clean section of paper towel, 100 μL of HRP-conjugated anti-mouse antibody were added to each well and incubated at 37°C for 30 minutes. Plates were washed six times with Tris buffered saline with Tween 20 (pH 7.6). Substrate solution was then added to each well, incubated at room temperature for 10 minutes, after which the optical density was read using a microplate reader set at 630 nm.

### Statistical analysis

All data are presented as means ± SD with SPSS13.0 software (SPSS, Chicago, IL, USA). Statistical analyses were performed by two-way analysis of variance (ANOVA) followed by S-N-K posthoc tests individually. The level of significance was set at P < 0.05.

## Competing interests

The authors declare that they have no competing interests.

## Authors' contributions

JL, XY, CZ, LM, JW, JS, and SX carried out the experiments and wrote the manuscript. SC, JW, and HA conceived the studies and participated in experimental design and coordination. All authors read and approved the final manuscript.
